# Serotype Is Associated With High Rate of Colistin Resistance Among Clinical Isolates of *Salmonella*

**DOI:** 10.3389/fmicb.2020.592146

**Published:** 2020-12-18

**Authors:** Qixia Luo, Yuan Wang, Hao Fu, Xiao Yu, Beiwen Zheng, Yunbo Chen, Björn Berglund, Yonghong Xiao

**Affiliations:** ^1^State Key Laboratory for Diagnosis and Treatment of Infectious Diseases, National Clinical Research Center for Infectious Diseases, The First Affiliated Hospital, College of Medicine, Zhejiang University, Hangzhou, China; ^2^Department of Clinical and Experimental Medicine, Linköping University, Linköping, Sweden

**Keywords:** *Salmonella* enterica, serotype, colistin susceptibility, clinical isolates, phylogenetic analysis, *pmr* genes

## Abstract

To investigate the prevalence, probable mechanisms and serotype correlation of colistin resistance in clinical isolates of *Salmonella* from patients in China, *Salmonella* isolates were collected from fecal and blood samples of patients. In this study, 42.8% (136/318) clinical isolated *Salmonella* were resistant to colistin. MIC distribution for colistin at serotype level among the two most prevalent serotypes originating from humans in China indicated that *Salmonella* Enteritidis (83.9% resistance, 125/149) were significantly less susceptible than *Salmonella* Typhimurium (15.3% resistance, 9/59, *P* < 0.01). *mcr* genes and mutations in PmrAB confer little for rate of colistin resistant *Salmonella* isolated from human patients. Phylogenetic tree based on core-genome single nucleotide polymorphisms (SNPs) was separately by the serotypes and implied a diffused distribution of MICs in the same serotype isolates. Relatvie expression levels of colistin resistant related *pmr* genes were significantly higher in non-*mcr* colistin resistant *S.* Typhimurium than in colistin sensitive *S.* Typhimurium, but no discernable differences between colistin resistant and sensitive *S.* Enteritidis, indicating a different mechanism between colistin resistant *S.* Typhimurium and *S.* Enteritidis. In conclusion, colistin susceptibility and colistin resistant mechanism of clinical isolated *Salmonella* were closely associated with specific serotypes, at least in the two most prevalent serotype Enteritidis and Typhimurium. We suggest clinical microbiology laboratory interpreting *Salmonella* colistin MIC results in the serotype level.

## Introduction

Polymyxins, which include polymyxin B and colistin, are cyclic polypeptide antibiotics that are synthesized by members of the *Paenibacillus* genus (Olaitan et al., 2014). As an old class of antimicrobials, polymyxins have been increasingly revitalized as a last resort drug to combat infections caused by multidrug-resistant (MDR) and carbapenem-resistant bacteria (Poirel et al., 2017). The target of polymyxins is the outer membrane lipopolysaccharide (LPS) of Gram-negative bacteria. Binding of polymyxin to the LPS increases the permeability of the bacterial membrane, leading to leakage of the cytoplasmic content and ultimately cell death (Olaitan et al., 2014). Resistance to polymyxins can be conferred via mutations in chromosomal genes, such as genes involved in the PmrAB/PhoPQ two-component systems, which promote the expression of LPS modification related genes, such as *pmrC*, *pmrE*, and *pmrHFJKLM* operon (Olaitan et al., 2014; Poirel et al., 2017). Plasmid-mediated colistin resistance genes (*mcr*) have since discovery in 2015 been frequently observed, and their continued widespread dissemination has became a challenge to public health worldwide (Liu et al., 2016; Luo et al., 2020).

*Salmonella* is one of the most important causes of foodborne diarrheal disease, even bloodstream infection (Ashton et al., 2017; Mohan et al., 2019). Although colistin is not a standardized option for treating infections caused by *Salmonella* in humans, the increasing reports of MDR and carbapenem-resistant *Salmonella*, in particular strains harboring *mcr*-genes isolated from patients (Kock et al., 2018; Lu et al., 2019; Sun et al., 2020), indicate that these pathogens constitute a public health concern which calls more attention to the epidemic characteristics of colistin resistance. Colistin resistance in *Salmonella* spp. can be conferred by mutations in *pmrAB*, or more rarely, by the colistin resistance gene *mcr* (Cui et al., 2017). Interestingly, our daily experimental results indicated the rate of colistin resistance among clinical isolates of *Salmonella* spp. are considerably higher compared to other Enterobacteriaceae, such as *Escherichia coli* and *Klebsiella pneumoniae* (Zhang et al., 2017). The objective of this study was to investigate the prevalence and probable mechanisms of colistin resistance in clinical isolates of *Salmonella* in China.

## Materials and Methods

### Collection of *Salmonella* Isolates and Serotyping

A total of 318 isolates of *Salmonella* spp. were collected from clinical samples nationwide in China from 2014 to 2018, including 218 isolates from bloodstream infection and 100 isolates from fecal samples that were derived from diarrhea patients (Supplementary Table 1). *Salmonella* serotyping was conducted according to the White–Kauffmann–Le Minor scheme (9th Edition) by performing a slide agglutination test (State Serum Institute (SSI), Copenhagen, Denmark).

### Colistin Susceptibility Testing

Cation-adjusted Mueller-Hinton Broth (Oxoid, Basingstoke, United Kingdom) dilutions were used for colistin MIC determination according to the CLSI-EUCAST joint recommendations. *E. coli* ATCC 25922 was used as a control. The experiment was conducted in duplicate on at least two separate occasions. The higher MIC was accepted for analysis in the duplicative test within one double dilution difference, or a third replicate would be measured (Kulengowski et al., 2019). The results were interpreted according to the EUCAST colistin breakpoint for Enterobacteriaceae (MIC > 2 mg/L, resistant).

### Anlaysis of Colistin Resistance Mechanisms

The isolates were screened by using primers targeting *mcr-1* to *mcr-9* from previous studies (Luo et al., 2017; Borowiak et al., 2020). For the colistin-resistant isolates, *pmrA* and *pmrB* were amplified by primers *pmrAB*-F and *pmrAB*-R (Supplementary Table 2). The positive PCR products were sequenced with Sanger sequencing for verification. The sequences of *pmrA* and *pmrB* were compared to that of *S.* Typhimurium LT2 (GenBank: GCA_000006945.2), a colistin-susceptible *Salmonella* reference strain.

### Whole-Genome Sequencing and Bioinformatic Analysis

Genomic DNA of 136 randomly selected *Salmonella* spp. isolates (including 64 colistin resistant and 72 susceptible isolates) were extracted using Gentra Puregene Yeast/Bact. Kit (Qiaqen, Hilden, Germany). Genomes were sequenced using the Illumina HiSeq 2500-PE150 platform (Illumina, San Diego, CA, United States). Quality-trimmed raw sequence data was assembled by using SPAdes 3.13.0. Annotation was performed by uploading the data to the RAST server (rast.nmpdr.org). All the assembled genomes were deposited in NCBI, the genome accession numbers were listed in Supplementary Table 1.

A phylogenetic tree based on SNPs in the core-genome was constructed via kSNP version 3.0 (Gardner et al., 2015). iTOL (V4) was used for the display, manipulation and annotation of phylogenetic trees (Letunic and Bork, 2019). Multi-sequence typing (MLST) were identified using BacWGSTdb (Ruan and Feng, 2016). All whole-genome sequenced *Salmonella* genomes were analyzed by using the SeqSero 1.2 software (Zhang et al., 2015)^1^ for serotype prediction. The results from SeqSero were compared to the traditional Kauffman-White serotyping. When the serotype of one isolate from SeqSero 1.2 was inconsistent with the serotyping from the slide agglutination test, the serotype was determined according to the phylogenetic clusters using core-genome SNPs.

### Reverse Transcription-Quantitative PCR (qRT-PCR)

Overnight cultures of the target *Salmonella* isolates were diluted 1:100 and subcultured in MH medium for ∼4 h at 37°C (OD_600_ ∼0.6). Cells were collected at 4°C by centrifuging at 10,000 rpm for 1 min, and RNA was extracted using TRIzol Reagent (Invitrogen). DNase I-treated RNA was obtained using an RNeasy Mini Kit (QIAGEN, No. 75142), and mRNA expression levels of the target genes were examined using real-time PCR primers listed in Supplementary Table 2. qRT-PCR was performed using an ABI 7300 96-well system (Applied Biosystems) with SYBR Premix Ex Taq II (cat. no. RR820A; TaKaRa). Expression levels of target genes were normalized against the 16S rRNA gene of *S. enterica* using the standard curve method.

### Statistical Analysis

Hypothesis testing was performed by stratifying the analyses for isolates of *Salmonella* Enteritidis and *Salmonella* Typhimurium. Due to the low prevalence of isolates with other serotypes, these were excluded from the analyses. Fisher’s exact tests were used to determine if resistant and susceptible phenotypes were equally distributed in *S.* Enteritidis and *S*. Typhimurium, and among isolates from blood and intestinal samples. A Mann-Whitney test was performed to determine if MICs were equally distributed among isolates of *S*. Enteritidis and *S*. Typhimurium. For the statistical analysis of relative expression levels of *pmr* genes, values are presented as means ± standard deviation (SD). Rank-sum tests were performed for pair-wise comparisons of groups, and *P* < 0.05 (two-tailed) was considered significant.

## Results

### Colistin MICs and Resistance Mechanisms Among *Salmonella* Isolated From Human Patients

**FIGURE 1:**
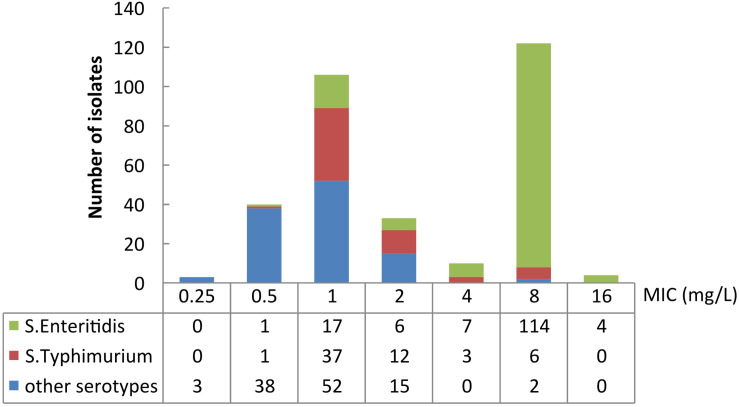
Number of isolates and MIC distribution of different serotypes of *Salmonella*. “Other serotypes” presents serotypes except for Enteritidis and Typhimurium.

The colistin MICs of the tested *Salmonella* spp. isolates ranged from 0.25 mg/L to 16 mg/L (Figure 1). The two most prevalent MICs were 8 mg/L (38.4%, 122/318) and 1 mg/L (33.3%, 106/318). 42.8% (136/318) of colistin MICs of the isolates were measured as > 2 mg/L. MIC_50_ and MIC_90_ of the isolates were 2 and 8 mg/L, respectively. 48.6% (106/218) of the bloodstream infection isolates were identified as colistin-resistant, whereas 30% (30/100) of the isolates from intestinal samples were determined as colistin-resistant.

Resistance mechanisms of all the colistin-resistant isolates were investigated by screening for *mcr* genes and sequencing of *pmrA* and *pmrB*. Out of 136 colistin-resistant isolates, two were as *mcr-1* positive (S039-44712 and S040-44862), none had other *mcr* genes, and two had *pmrAB* mutations which affected the amino acid sequence (S132-54197 and S133-54550). The isolates with *pmrAB* mutations carried a missense point mutation in *pmrA* (T89S), and five missense mutations in *pmrB* (M15T, G73S, V74I, I83V, A111T). However, none of the mutations were predicted to affect the protein function.

### Relation Between Serotypes and Colistin Susceptibility

18 serotypes were identified among the 318 isolates. *S.* Enteritidis (*n* = 149) and *S.* Typhimurium (*n* = 59) were found to be the overall most prevalent. *S.* Enteritidis (122/218) was the most common among isolates from bloodstream infection samples, followed by *S.* Typhimurium (12/218), whereas the inverse was true among isolates from patients with intestinal infections, with 27.0% (27/100) *S.* Enteritidis and 47.0% (47/100) *S.* Typhimurium (*P* < 0.01).

The rate of colistin-resistant *S.* Enteritidis and *S.* Typhimurium were 83.9% (125/149) and 15.3% (9/59), respectively. Isolates of *S.* Enteritidis were significantly less susceptible than isolates of *S.* Typhimurium (*P* < 0.01) and had significantly higher MICs (*P* < 0.01). *S.* Enteritidis was most prevalent among the colistin-resistant isolates, representing 91.9% (125/136) of all with the resistance phenotype, followed by *S.* Typhimurium (6.6%, 9/136). All colistin-resistant isolates were *S.* Enteritidis and *S.* Typhimurium, except for the two isolates (S132-54197 and S133-54550) which were identified as *Salmonella* Gallinarum (1/2) and *Salmonella* Goldcoast (1/1), respectively.

### Serotype Comparison and Phylogenetic Analysis

In order to discover the relation between *Salmonella* serotypes, phylogenetic evolution and MICs, *Salmonella* serotypes were confirmed and phylogenetic trees based on core-genome SNPs were generated. The whole-genome sequenced isolates were predicted serotypes using SeqSero 1.2 and were compared to the traditional Kauffman-White serotyping. Phylogenetic analysis was employed to confirm the serotyping, by using core-genome SNPs as described above. 63 *S.* Enteritidis, 45 *S*. Typhimurium and 28 isolates of other serotypes were identified from the 136 whole-genome sequenced isolates, and were 98.4% (63/64), 97.8% (45/46), and 92.9% (28/30) identical to the traditional Kauffman-White serotyping, respectively.

**FIGURE 2:**
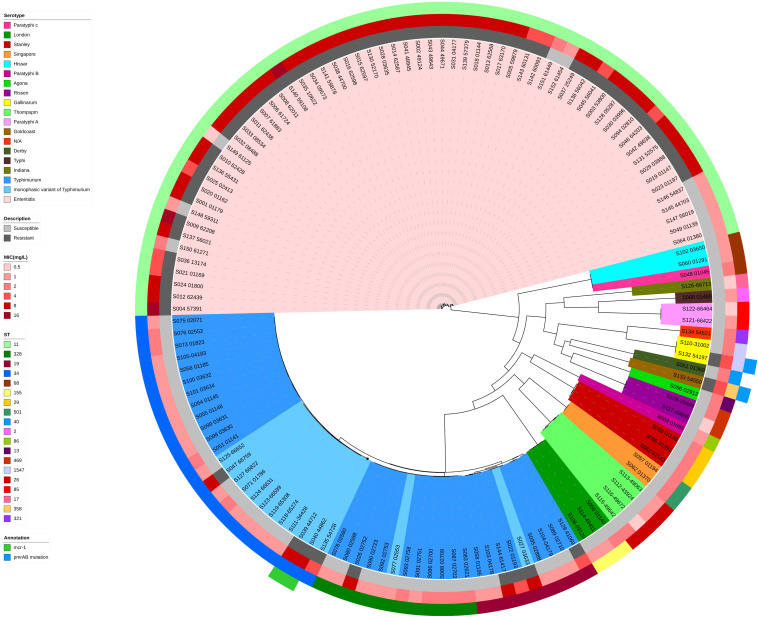
Maximum parsimony tree of the whole-genome sequenced 136 clinical *Salmonella* isolates. MICs (mg/L) and sequence types of colistin among different serotype *Salmonella* isolates were shown. Maximum parsimony tree were generated based on core-genome SNPs by kSNP 3.0. Isolates with different serotypes are indicated in the inner ring with different colors. Colistin MICs in mg/L are shown, along with corresponding *mcr-1* gene and *pmrAB* mutations known to be associated with phenotypic resistance. For the description of colistin susceptibility, resistance corresponds to MIC > 2 mg/L and susceptible corresponds to MIC ≤ 2 mg/L.

**FIGURE 3:**
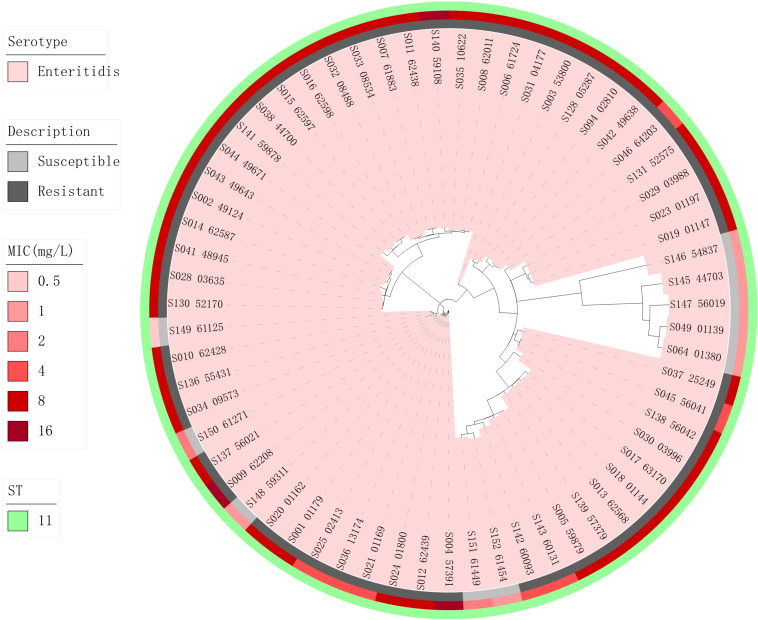
Zoomed-in maximum parsimony tree showing the Enteritidis serotype sub-clade of the clinical *Salmonella* isolates. Colistin MICs in mg/L and sequence types are shown. For the description of colistin susceptibility, resistance corresponds to MIC > 2 mg/L and susceptible corresponds to MIC ≤ 2 mg/L.

**FIGURE 4:**
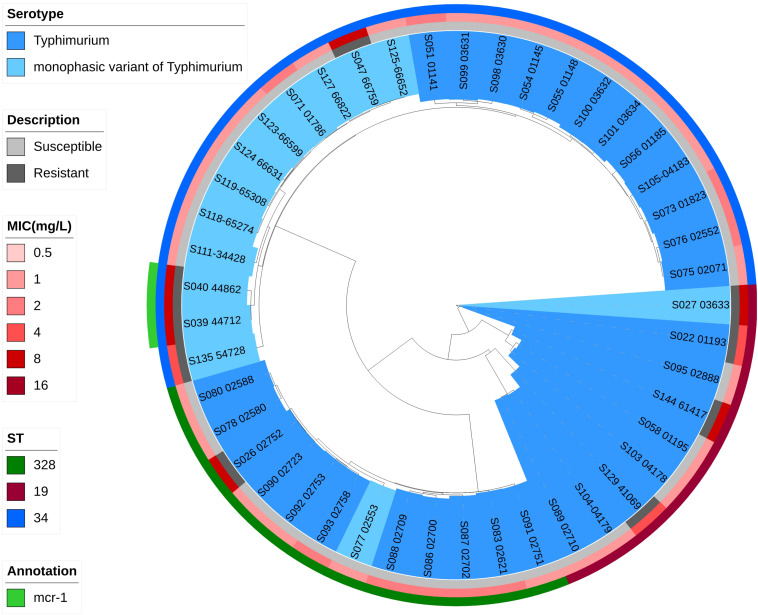
Zoomed-in maximum parsimony tree showing the Typhimurium and monophasic variant of Typhimurium (*Salmonella* 4,[5],12:i:-) serotypes sub-clade of the clinical *Salmonella* isolates. Colistin MICs in mg/L and sequence types are shown. For the description of colistin susceptibility, resistance corresponds to MIC > 2 mg/L and susceptible corresponds to MIC ≤ 2 mg/L.

Phylogenetic trees based on core-genome SNPs were generated for WGS isolates of all serotypes (Figure 2), for all *S.* Enteritidis isolates (Figure 3) and for all *S.* Typhimurium isolates (Figure 4). The relation between serotypes, sequence types and MICs was inferred in the phylogenetic tree (Figure 2). The isolates clustered by serotypes and sequence types in the phylogenetic tree but not by MICs. The *S.* Enteritidis isolates showed little variation in their core genomes. The numbers of SNPs of serotype Enteritidis isolates ranged from 1 to 236, which was very closely in evolution, but did not constitute a single clone. Sequence type of all serotype Enteritidis were ST11 (Figure 3). Five colistin-susceptible isolates (S049, S064, S145, S146, S147) clustered together while the remaining five colistin-susceptible isolates (S148, S149, S150, S151, S152) were more diffusely distributed (Figure 3). In contrast, isolates of *S.* Typhimurium showed a considerable diversity in their core genomes (Figure 4). The two *mcr-1* positive *S*. Typhimurium isolates were both monophasic variant of Typhimurium and clustered together, while the other colistin-resistant *S*. Typhimurium isolates were more dispersed.

### Comparision of Expression Levels of *pmr* genes in Colistin Resistant and Sensitive *S.* Typhimurium or *S.* Enteritidis

**FIGURE 5:**
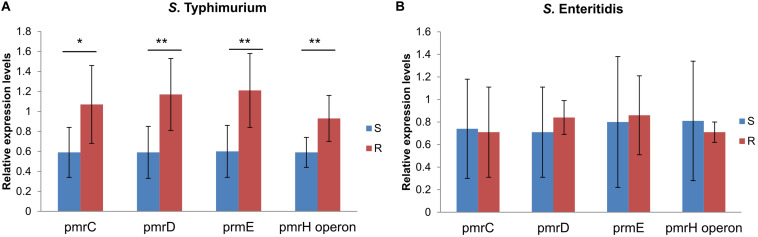
Relative expression levels of *pmrC*, *pmrD*, *pmrE* and *pmrHFJKLM* operon in colistin resistant and sensitive isolates of serotype Typhimurium **(A)** and Enteritidis **(B)**. Gene expression levels of each target gene were identified and compared with 16s rRNA expression levels of each isolates. Relative gene expression levels of colistin sensitive (S) group and colistin resistant (R) group in serotype Typhimurium and Enteritidis were compared using *p*-values calculated by Statistical Package for the Social Sciences (SPSS) using rank-sum tests, respectively (*P < 0.05, **P < 0.01).

In order to discover if there are different mechanisms between colistin resistant *S.* Typhimurium and *S.* Enteritidis, expression levels of LPS modification related *pmr* genes (*pmrC*, *pmrD*, *pmrE*, and *pmrHFJKLM* operon) in colistin sensitive *S.* Typhimurium or *S.* Enteritidis were compared with non-*mcr* colistin resistant *S.* Typhimurium or *S.* Enteritidis, respectively. Seven isolates of each serotype were colistin resistance (Supplementary Table 1) and seven were colistin sensitive (Supplementary Table 1). Relatvie expression levels of all *pmr* genes were enhanced in colistin resistant *S.* Typhimurium than in colistin sensitive *S.* Typhimurium (*P* < 0.05), but no discernable differences between colistin resistant and sensitive *S.* Enteritidis (Figure 5), indicating a different mechanism between colistin resistant *S.* Typhimurium and *S.* Enteritidis.

## Discussion

Previous studies on colistin susceptibility of *Salmonella* spp. utilizing surveillance data from poultry, food and human clinical sources in European countries and the United States, have indicated a correlation between *Salmonella* serotype and colistin MICs (Tyson et al., 2018; Alvarez et al., 2019). Though the occurrence of *Salmonella* serotypes vary from different countries and sources, the inhomogeneity was confirmed in the current study in which the high rate of colistin resistance of *Salmonella* spp. isolated from human patients from China was clearly linked to specific serotypes.

*S.* Enteritidis and *S.* Typhimurium were the predominant serotypes overall (with *S.* Enteritidis being the most prevalent), however, there was a sizeable and significant difference in colistin susceptibility and MICs between isolates of the two serotypes. Interestingly, the colistin resistance rate of bloodstream infection isolates was significantly higher compared to that of isolates from intestinal samples (*P* < 0.01). This difference was attributable to that *S.* Enteritidis were significantly more common among bloodstream isolates compared to the *S.* Typhimurium (*P* < 0.01). This indicates that not only is *S.* Enteritidis more likely to be colistin-resistant compared to *S.* Typhimurium, but also more frequently cause severe infections in China.

Carriage of *mcr* genes and missense mutations causing amino acid substitutions in PmrAB are the most commonly reported colistin resistance mechanisms in *Salmonella* spp. Surprisingly, in this study, only four isolates were observed with either of these resistance mechanisms, including two *S.* Typhimurium isolates carried *mcr-1* and two isolates had missense mutations in *pmrAB.* According to previous studies in China, *S.* Typhimurium appear to more easily acquire *mcr* genes compared to *S.* Enteritidis (Li et al., 2016; Cui et al., 2017; Luo et al., 2020). This may be because *S.* Typhimurium in general is more susceptible to colistin than *S.* Enteritidis, and so tend to acquire *mcr* genes in order to adapt to colistin selection pressure. No *pmrAB* mutations were found in colistin resistant *S*. Enteritidis, though the high rate of colistin resistance were observed in this serotype. Relatvie expression levels of *pmr* genes were enhanced in colistin resistant *S.* Typhimurium than in colistin sensitive *S.* Typhimurium, but no discernable differences between colistin resistant and sensitive *S.* Enteritidis, which indicating a *pmr* related mechanism that confers to colistin resistance to non-*mcr S.* Typhimurium, but a *pmr* unrelated mechanism in colistin resistant *S.* Enteritidis. The large proportion of isolates with unknown resistance mechanisms in the current study indicate that other, as of yet uncharacterized, resistance mechanisms may be more important for *Salmonella* spp.

Aside from that unknown colistin resistance mechanisms could account for the difference in colistin resistance rates of *S.* Enteritidis and *S.* Typhimurium in this study, there could also be intrinsic differences between the serotypes which play an important role. The target of polymyxins is the outer membrane LPS of Gram-negative bacteria. These suggests that the O-antigen (surface LPS of the cell) might play a role in colistin susceptibility. Whether and how different O-antigen confer a different colistin susceptibility phenotype of *Salmonella* needs to be further investigation.

An epidemiological cut-off value for colistin regarding *Salmonella* spp. is lacking in the data presented by EUCAST; currently, only the MIC distribution of *S.* Dublin is presented. The results in this study indicate a very high rate of colistin resistance among *S.* Enteritidis in clinical isolates in China, and this could be associated to elevated wild type MICs compared to other serotypes. Further studies including isolates of *S.* Enteritidis from other countries and other sources are warranted to explore this possibility.

## Data Availability Statement

The original contributions presented in the study are publicly available. This data can be found in NCBI under accession Nos. WPIS00000000-WPNX00000000.

## Ethics Statement

The studies involving human participants were reviewed and approved by the Ethics Committee of the First Affilated Hospital of Zhejiang University. The patients/participants provided their written informed consent to participate in this study.

## Author Contributions

QL and YX conceived and designed the experiments. YW, HF, XY, and YC performed the experiments. QL, BB, and BZ analyzed the data. QL wrote the manuscript. All authors contributed to the article and approved the submitted version.

## Conflict of Interest

The authors declare that the research was conducted in the absence of any commercial or financial relationships that could be construed as a potential conflict of interest.

## Footnotes

1 https://cge.cbs.dtu.dk/services/SeqSero/
