# Supplementary material for: Serotype Is Associated With High Rate of Colistin Resistance Among Clinical Isolates of Salmonella
Source: Front Microbiol. 2020 Dec 18;11:592146. doi: 10.3389/fmicb.2020.592146 (PMC7775366; doi:10.3389/fmicb.2020.592146)
Supplement: Supplementary file 2 [file Table_2.docx]

**Supplementary materials**

**Serotype is associated with high rate of colistin resistance among clinical isolates of *Salmonella***

Qixia Luo^1^, Yuan Wang^1^, Hao Fu^1^, Beiwen Zheng^1^, Xiao Yu^1^, Yunbo Chen^1^, Björn Berglund^1,2^, Yonghong Xiao^1*^.

^1^State Key Laboratory for Diagnosis and Treatment of Infectious Diseases, National Clinical Research Center for Infectious Diseases, The First Affiliated Hospital, College of Medicine, Zhejiang University, Hangzhou, China.

^2^Department of Clinical and Experimental Medicine, Linköping University, Linköping, Sweden.

***Corresponding author**

Address: The First Affiliated Hospital, College of Medicine, Zhejiang University, Qingchun Road 79, 310003, Hangzhou, China.

Telephone: +86-571-87236427

Fax: +86-571-87236427
E-mail addresses: [xiaoyonghong@zju.edu.cn](mailto:xiaoyonghong@zju.edu.cn)

**Supplementary Table**

**Table S2.** qRT**-**PCR primers used in this study.

| Primer name | Sequence | Product |
| --- | --- | --- |
| *mcr*-1-F | CGGTCAGTCCGTTTGTTC | 309 bp |
| *mcr*-1-R | CTTGGTCGGTCTGTAGGG |  |
| *mcr*-2-F | CAAGTGTGTTGGTCGCAGTT | 700 bp |
| *mcr*-2-R | TCTAGCCCGACAAGCATACC |  |
| *mcr*-3-F | AAATAAAAATTGTTCCGCTTATG | 900 bp |
| *mcr*-3-R | AATGGAGATCCCCGTTTTT |  |
| *mcr*-4-F | TCACTTTCATCACTGCGTTG | 1100 bp |
| *mcr*-4-R | TTGGTCCATGACTACCAATG |  |
| *mcr*-5-F | ATGCGGTTGTCTGCATTTATC | 1644 bp |
| *mcr*-5-R | TCATTGTGGTTGTCCTTTTCTG |  |
| *mcr-*6-F | AGCTATGTCAATCCCGTGAT | 252 bp |
| *mcr-*6-R | ATTGGCTAGGTTGTCAATC |  |
| *mcr*-7-F | GCCCTTCTTTTCGTTGTT | 551 bp |
| *mcr*-7-R | GGTTGGTCTCTTTCTCGT |  |
| *mcr*-8-F | TCAACAATTCTACAAAGCGTG | 856 bp |
| *mcr*-8-R | AATGCTGCGCGAATGAAG |  |
| *mcr*-9-F | TTCCCTTTGTTCTGGTTG | 1011 bp |
| *mcr*-9-R | GCAGGTAATAAGTCGGTC |  |
| *pmrAB-F* | CAGGAGACTAAGCGAATGAA | 1949 bp |
| *pmrAB-R* | GTGCTGTTGAAAAAGGCATAA |  |
| Sal-*pmrD*-RT-F | AAGAGGGCGTGCCATGTTCT | 131 bp |
| Sal*-pmrD*-RT-R | ATACAATACTGCGCATCCTG |  |
| Sal-*pmrC*-RT-F | TGCGTTCTACAAGCAGGTAC | 174 bp |
| Sal-*pmrC*-RT-R | GCAGCGCCGACCAGAATAAA |  |
| Sal-*pmrE*-RT-F | CTTCCCGTGTTGAACTGTTA | 275 bp |
| Sal-*pmrE*-RT-R | TTGGGATCGTAATCCGTAGG |  |
| Sal-*pmrH*-RT-F | AGGTTGGATAACAACCGGCC | 157 bp |
| Sal*-pmrH*-RT-R | TGTAATGACCTCGTCGCCTT |  |
| Sal-16s-RT-F | CGGGGAGGAAGGTGTTGTG | 178 bp |
| Sal-16s-RT-R | GAGCCCGGGGATTTCACATC |  |
